# Non-destructive Assessment of Plant Nitrogen Parameters Using Leaf Chlorophyll Measurements in Rice

**DOI:** 10.3389/fpls.2016.01829

**Published:** 2016-12-15

**Authors:** Syed Tahir Ata-Ul-Karim, Qiang Cao, Yan Zhu, Liang Tang, Muhammad Ishaq Asif Rehmani, Weixing Cao

**Affiliations:** ^1^National Engineering and Technology Center for Information Agriculture, Jiangsu Key Laboratory for Information Agriculture, Jiangsu Collaborative Innovation Center for Modern Crop Production, Nanjing Agricultural UniversityNanjing, China; ^2^Department of Agronomy, Ghazi UniversityDera Ghazi Khan, Pakistan

**Keywords:** chlorophyll meter, critical nitrogen, nitrogen nutrition index, accumulated nitrogen deficit, nitrogen requirement, phenological stages, rice

## Abstract

Non-destructive assessment of plant nitrogen (N) status is essential for efficient crop production and N management in intensive rice (*Oryza sativa* L.) cropping systems. Chlorophyll meter (SPAD-502) has been widely used as a rapid, non-destructive and cost-effective diagnostic tool for in-season assessment of crop N status. The present study was intended to establish the quantitative relationships between chlorophyll meters readings, plant N concentration (PNC), N nutrition index (NNI), accumulated N deficit (AND), and N requirement (NR), as well as to compare the stability of these relationships at different vegetative growth stages in Japonica and Indica rice cultivars. Seven multi-locational field experiments using varied N rates and seven rice cultivars were conducted in east China. The results showed that the PNC and chlorophyll meters readings increased with increasing N application rates across the cultivars, growing seasons, and sites. The PNC and chlorophyll meters readings under varied N rates ranged from 2.29 to 3.21, 1.06 to 1.82 and 37.10 to 45.4 and 37.30 to 46.6, respectively, at TL and HD stages for Japonica rice cultivars, while they ranged from 2.25 to 3.23, 1.34 to 1.91 and 35.6 to 43.3 and 37.3 to 45.5 for Indica rice cultivars, respectively. The quantitative relationships between chlorophyll meters readings, PNC, NNI, AND, and NR established at different crop growth stages in two rice ecotypes, were highly significant with *R*^2^ values ranging from 0.69 to 0.93 and 0.71 to 0.86 for Japonica and Indica rice, respectively. The strongest relationships were observed for AND and NR at panicle initiation and booting stages in both rice ecotypes. The validation of the relationships developed in the present study with an independent data exhibited a solid model performance and confirmed their robustness as a reliable and rapid diagnostic tool for in-season estimation of plant N parameters for sustainable N management in rice. The results of this study will offer a suitable approach for managing N application precisely during the growth period of the rice crop in intensive rice cropping systems of east China.

## Introduction

Nitrogen (N) is the most essential nutrients imperative for crop growth and productivity. Effective in-season N management, both in terms of rate and timing of application is vital for improving crop productivity and quality as well as for sustaining soil fertility in rice (*Oryza sativa* L.) cropping systems (Ata-Ul-Karim et al., 2016). N is an indispensable constituent of chlorophyll and protein associated with leaf color, plant vigor, plant N status, yield and quality (Adhikari et al., 1999). Insufficient N application leads to smaller leaves, lower chlorophyll and protein content, lower dry matter (DM) accumulation and leaf area expansion in rice (Ata-Ul-Karim et al., 2013, 2014b; Wang et al., 2014). Therefore, N is often applied in excess in order to enhance rice crop productivity, especially in intensive rice cropping systems of China (Peng et al., 2009, 2010). The excessive N application is not only the major cause of the lower N use efficiency in rice cultivation of China (Peng et al., 1996, 2006) but also responsible for creating hazards for health, environmental sustainability and biodiversity (Liu et al., 2013; Bodirsky et al., 2014). The aforementioned issues have stirred much research effort aiming to develop efficient and sustainable N management strategies for improving the N use efficiency of the rice crop and environmental sustainability, especially in China.

Chlorophyll meter (SPAD-502), being a simple, swift, and non-destructive method, has been extensively used technique for estimating plant N status. However, several factors such as plant growth stage, cultivar, specific leaf weight, leaf thickness, leaf position on the plant, measurement location on a leaf (Muñoz-Huerta et al., 2013; Yuan et al., 2016b) as well as environmental stresses and solar radiation (Zhao et al., 2016a) could significantly affect chlorophyll meter readings. Effective measures such as correlating the SPAD values and leaf N concentration (Esfahani et al., 2008), developing SPAD sufficiency index (Hussain et al., 2000), relative SPAD index (Ziadi et al., 2008; Yuan et al., 2016a), normalized SPAD index (Debaeke et al., 2006; Yuan et al., 2016a), positional difference SPAD indices (Lin et al., 2010; Yuan et al., 2016a; Zhao et al., 2016b), and the relationships between chlorophyll meter readings and canopy color related images obtained by a digital still color camera (Wang et al., 2014) have been widely adopted by researchers to minimize the influences of aforementioned factors on chlorophyll meter readings. Chlorophyll meter readings are well correlated with leaf N concentration, color-related indices from digital still camera, chlorophyll content and soil mineral N concentration (Thind and Gupta, 2010; Wang et al., 2014), yet regulating chlorophyll meter readings to direct units of PNC is still challenging and requires a comprehensive understanding of the relationship between chlorophyll meter readings and PNC across the phenological stages, cultivars, and sites.

Chlorophyll meter readings must be evaluated against the well-established diagnostic techniques for their reliable and efficient implementation in the field. The concept of critical N is based on actual crop growth and is crop specific, precise, simple and biologically sound technique and has been widely used for in-season N diagnosis in various crop species including rice (Ata-Ul-Karim et al., 2013, 2014a,b; Yao et al., 2014). Yet it is destructive, expensive and time-consuming, and also poses negative environmental impacts due to the production of noxious chemicals during analysis. Therefore, the integrated investigation of chlorophyll meter readings with the concept of critical N curve and critical N based parameters [N nutrition index (NNI), accumulated N deficit (AND) and N requirement (NR)] may give a better and more robust insight into the underlying mechanism of N uptake and effective diagnosis in rice crops. Attempts have been made to relate the chlorophyll meter readings with PNC and NNI using several indirect methodologies. These include relative chlorophyll meter readings (Ziadi et al., 2008; Yuan et al., 2016a), normalized chlorophyll meter readings (Debaeke et al., 2006; Yuan et al., 2016a), chlorophyll meter readings of different leaf position (Prost and Jeuffroy, 2007; Yang et al., 2014; Yuan et al., 2016a; Zhao et al., 2016b) and positional difference indices for estimating crop N status (Yuan et al., 2016a; Zhao et al., 2016b). Despite these attempts, there remains controversy regarding their reliability in estimating plant N status due to contradiction regarding the effect of different leaf positions on chlorophyll meter readings, which affects the reliability of these techniques in estimating plant N status. We hypothesized that the averaged chlorophyll meter readings value of top four leaves can minimize the influences of leaf position on the plant and sampling location on the leaf on chlorophyll meter readings. Attempts have been made to relate averaged values of chlorophyll meter readings with leaf N concentration and image color indices obtained by digital still camera (Wang et al., 2014), yet no recent studies have been reported to assess plant N status by integrating the chlorophyll meter readings with PNC, NNI, AND, and NR at different growth stages in agronomic crops, including different rice ecotypes.

Therefore, the present study aimed to establish the integrated and quantitative relationships between chlorophyll meter readings, PNC, NNI, AND, and NR at different growth stages of Japonica and Indica rice, and to validate the suitability of these relationships to overcome the aforementioned inadequacies for simpler and non-destructive estimation of plant N status during rice production. The integration of the aforementioned techniques will be helpful in developing a promising precision N management approach in rice. The projected results would help to develop a simple, rapid, non-destructive and cost-effective N management strategy at different phenological stages in rice.

## Materials and Methods

### Experimental Design

Seven field experiments were conducted at three sites: Yizheng (32°16′ N, 119° 10′ E), Rugao (32°23′ N, 120° 33′ E) and Wujiang (31°15′ N, 120°72′ E) situated in east China during 2010–2014. Rice was grown under a rage on N rates (0–375 kg N ha^-1^, **Table 1**) in each experiment. The five Japonica rice including Lingxiangyou18 (LXY18), Wuxiangjing14 (WXJ14), Wuyunjing24 (WYJ24), Wuyunjing19 (WYJ19); and Yongyou8 (YY8) and two Indica rice, Shanyou63 (SY63) and YLiangyou1 (YLY1) cultivars transplanted in field were seven of the most widely cultivated rice cultivars in the middle and lower Yangtze River Reaches. The experiments were arranged is a randomized complete block design with three replications in all the experiments. The size of each plot at Yizheng (Experiments 1–3) was 4.5 m × 8 m while in Rugao and Wujiang it was 5 m × 6 m (Experiments 4–7). The hill spacing of 0.30 m × 0.15 m, with two seedlings per hill was used at all the sites, resulting in a planting density of approximately 22.2 × 10^4^ plants ha^-1^. In each experiment, 59 kg ha^-1^ phosphorus as P_2_O_5_ and 158 kg ha^-1^ potassium as K_2_O were incorporated in each plot before transplantation. Crop management practices at each site were performed according to local recommendations in order to achieve the potential grain yield (only limiting factor was N fertilizer application). Weeds, diseases, and insects were intensively controlled throughout the growing period. **Table 1** gives a summary of the detailed information about rice cultivars used in each experiment and N treatments in seven field experiments including N application rates, the amount of N applied at the different growth stages, along with soil properties.

**Table 1:** Basic information about seven field experiments involving varied N rates in Japonica (J) and Indica (I) rice cultivars conducted during 2010–2014.

Experiment No.	Soil property	Cultivar	N rate (kg ha^-1^)	N distribution (%) and stages	Sampling stage	Sampling date
						
Experiment 1 (2010) Yizheng	Soil pH: 6.2	LXY18 (J)	0 (N0)	PP (50%)	MT	17-July
	Organic matter:17.5 g kg^-1^	WXJ14 (J)	80 (N1)	AT (10%)	SE	26-July
	Total N: 1.6 g kg^-1^		160 (N2)	PI (20%)	PI	8-August
	Available P: 43 mg g^-1^		240 (N3)	BT (20%)	BT	20-August
	Available K: 90 mg g^-1^		320 (N4)		HD	30-August
Experiment 2 (2011) Yizheng	Soil pH: 6.4	LXY18 (J)	0 (N0)	PP (50%)	MT	21-July
	Organic matter: 15.5 g kg^-1^	WXJ14 (J)	90 (N1)	AT (10%)	SE	26-July
	Total N: 1.3 g kg^-1^		180 (N2)	PI (20%)	PI	2-August
	Available P: 38 mg g^-1^		270 (N3)	BT (20%)	BT	25-August
	Available K: 85 mg g^-1^		360 (N4)		HD	3-September
Experiment 3 (2011) Yizheng	Soil pH: 6	SY63 (I)	0 (N0)	PP (50%)	MT	21-July
	Organic matter: 19.2 g kg^-1^		70 (N1)	AT (10%)	SE	30-July
	Total N: 1.7 g kg^-1^		170 (N2)	PI (20%)	PI	9-August
	Available P: 42 mg g^-1^		270 (N3)	BT (20%)	BT	18-August
	Available K: 95 mg g^-1^		370 (N4)		HD	30-August
Experiment 4 (2012) Rugao	Soil pH: 6	SY63 (I)	0 (N0)	PP (50%)	MT	20-July
	Organic matter: 13.5 g kg^-1^		70 (N1)	AT (10%)	SE	30-July
	Total N: 1.5 g kg^-1^		170 (N2)	PI (20%)	PI	9-August
	Available P: 30 mg g^-1^		270 (N3)	BT (20%)	BT	19-August
	Available K: 84 mg g^-1^		370 (N4)		HD	29-August
Experiment 5 (2013) Rugao	Soil pH: 6.1	WXJ14 (J)	0 (N0)	PP (40%)	MT	19-July
	Organic matter: 14.9 g kg^-1^	SY63 (I)	75 (N1)	AT (10%)	SE	29-July
	Total N: 1.1 g kg^-1^		150 (N2)	PI (30%)	PI	9-August
	Available P: 32 mg g^-1^		225 (N3)	BT (20%)	BT	19-August
	Available K: 80 mg g^-1^		300 (N4)		HD	28-August
			375 (N5)			
Experiment 6 (2013) Wujiang	Soil pH: 6.4	WYJ19 (J)	0 (N0)	PP (40%)	MT	18-July
	Organic matter: 36.5 g kg^-1^	YY8 (J)	90 (N1)	AT (10%)	SE	31-July
	Total N: 2.1 g kg^-1^		180 (N2)	PI (30%)	PI	13-August
	Available P: 45.5 mg g^-1^		270 (N3)	BT (20%)	BT	20-August
	Available K: 115.3 mg g^-1^		360 (N4)		HD	27-August
Experiment 7 (2014) Rugao	Soil pH: 6.4	WYJ24 (J)	0 (N0)	PP (40%)	MT	18-July
	Organic matter: 23 g kg^-1^	YLY1 (I)	150 (N1)	AT (10%)	SE	27-July
	Total N: 1.35 g kg^-1^		225 (N2)	PI (30%)	PI	6-August
	Available P: 46.2 mg g^-1^		300 (N3)	BT (20%)	BT	16-August
	Available K: 105.5 mg g^-1^		375 (N4)		HD	26-August


### Collection of Chlorophyll Meter Readings

Chlorophyll meter readings were obtained at five growth stages including tillering (TL), stem elongation (SE), panicle initiation (PI), booting (BT), and heading (HD) using a SPAD-502 meter (Minolta Camera Co., Osaka, Japan). Chlorophyll meter readings were measured from the four uppermost fully expanded leaves of ten randomly selected plants from each plot. Five evenly spaced chlorophyll meter readings, avoiding leaf midribs, were taken per leaf at each growth stage and averaged as the mean chlorophyll meter readings of the leaf. Then, the chlorophyll meter readings of top four leaves for every experimental plot were averaged to minimize the influences of leaf position on the plant and sampling location on the leaf on chlorophyll meter readings.

### Plant Sampling and Measurements

Five plant samples per plot were harvested from 0.23 m^2^ areas at different vegetative growth stages in all experiments to determine plant DM and PNC. The plant samples were divided into green leaf blade (leaf) and culm plus sheath (stem). All the samples were oven-dried for 30 min at 105°C to substantially reduce plant metabolic activities and then at 70°C to constant weight to attain the plant DM (t ha^-1^). The PNC (%) was determined by using the standard Kjeldahl method (Bremner and Mulvaney, 1982). The N accumulation (kg N ha^-1^) was calculated by multiplying plant DM by PNC.

### Determination of Critical N Parameters

#### Nitrogen Nutrition Index

The NNI, the ratio between the actual crop N concentration (Na) and critical N concentration (Nc), of different rice cultivars at different vegetative growth stages was calculated from Justes et al. (1994):

NNI=Na/Nc

#### Accumulated Nitrogen Deficit

The AND (kg N ha^-1^), which is the amount of N, which a crop fails to take up at any stage of development for reaching the level of critical N uptake (i.e., the deficit of crop N uptake which is required to achieve maximum yield) of different cultivars/ecotypes of rice at different growth stages was calculated according to the method proposed by Ata-Ul-Karim et al. (2013):

AND=Ncna-Nna

where Ncna is the N accumulation under the Nc growth condition, and Nna is the actual N accumulation under different N rates. Ncna can be obtained by multiplying the critical plant DM with critical N concentration at each growth stage.

#### Nitrogen Requirement

Nitrogen requirement (kg N ha^-1^), the N fertilizer requirement for a crop at any stage of development for reaching the N*_c_* level (i.e., the crop N status corresponding to maximum growth) of rice cultivars/ecotypes at different growth stages was calculated as (Ata-Ul-Karim et al., 2017):

$$\text{NR=}\left(\frac{\text{Ncna-Nna}}{\text{NRE}}\right)$$

where NRE is the N recovery use efficiency of in-season N fertilizer application.

The NRE, the ratio of plant N to N supply at various crop growth stages was calculated for different rice cultivars/ecotypes, years, and sites from Novoa and Loomis (1981):

$$\text{NRE=}\left(\frac{{\text{Nupt}}_{\text{fert}-{\text{Nupt}}_{\text{unfert}}}}{\Delta \text{N applied}}\right)$$

where Nupt_fert_ is N uptake in the fertilized plot, Nupt_unfert_ is the N uptake in the corresponding unfertilized plot and ΔN is N fertilizer applied.

The N uptake in the fertilized and unfertilized plot, as well as ΔN applied, was calculated according to the N distribution at each growth stage and year to calculate NRE at each growth stage.

### Statistical Analysis

The data of PNC, chlorophyll meter readings and plant DM at each sampling stage, year and rice ecotype obtained under the varied N rates were subjected to analysis of variance (ANOVA) using GLM procedures in IBM SPSS Version19.0 (IBM Corporation, Armonk, NY, USA). The differences between treatment means were assessed using least significant difference (LSD) test at 90% level of significance, instead of classically using 95% in order to reduce the occurrence of Type II errors (i.e., the error of incorrectly retaining a false null hypothesis). The linear regression relationships between chlorophyll meter readings and PNC NNI, AND, and NR were developed using Microsoft Excel 2010 (Microsoft Corporation, Redmond, WA, USA). Regression analyses were conducted for each growth stage of Japonica and Indica rice using the data collected in Experiments 1, 3, 4, and 7. The data sets used for developing these relationships were collected from Experiments 1, 3, 4, and 7, as these experiments represent different sites (Yizheng and Rugao), cultivars (LXY18, WXJ14, SY63, WYJ24, and YLY1) and ecotypes (Japonica and Indica). This selection was carried out with the objective of developing the reliable relationships.

### Validation of the Quantitative Relationships

The relationships between chlorophyll meter readings and PNC NNI, AND, and NR established at different growth stages were validated to check the consistency of the relationships for different sites and cultivars/ecotypes by using the data acquired from three independent experiments (Experiments 2, 5, and 6). These experiments represent different sites (Yizheng, Rugao, and Wujiang), cultivars (LXY18, WXJ14, SY63, WYJ19, and YY8) and ecotypes (Japonica and Indica). The root mean square error (RMSE) between the predicted and observed values was used to test the goodness of fit of the linear regression models between chlorophyll meter readings and PNC NNI, AND, and NR. RMSE was calculated as (Ata-Ul-Karim et al., 2016, 2017):

RMSE(%)=(1nΣi=1n(yi-y^i))2×100y¯

where yi, ŷi, y and n represent observed values estimated values, the mean of the observed values and number of samples, respectively.

## Results

### Changes in PNC Over Growth Stages under Varied N Rates in Japonica and Indica Rice

**FIGURE 1:**
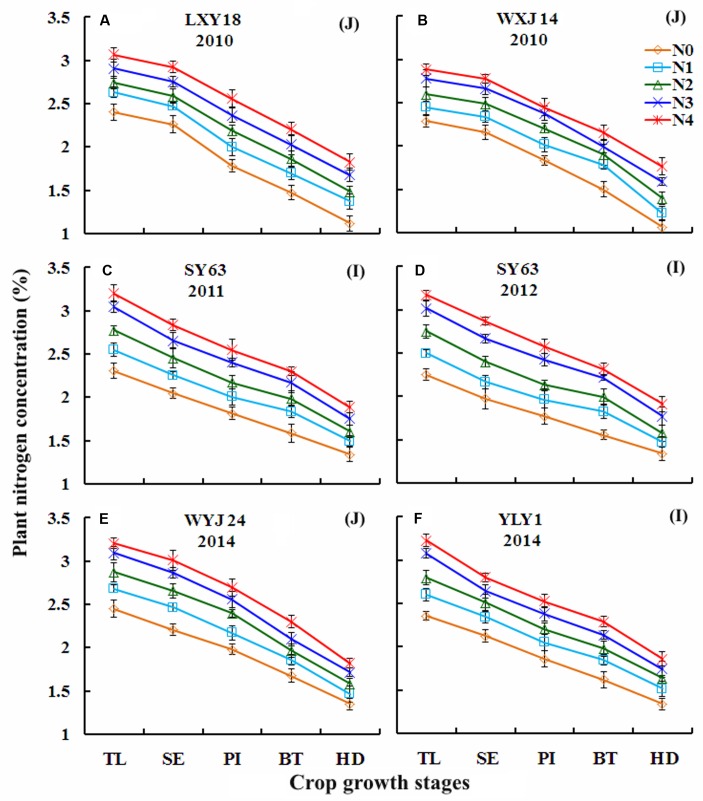
**Changes in plant nitrogen concentration (PNC) over growth progress under varied N rates in Japonica (J) and Indica (I) rice cultivars during 2010–2014.** **(A)** LXY18, 2010; **(B)** WXJ14, 2010; **(C)** SY63, 2011; **(D)** SY63, 2012; **(E)** WYJ24, 2014; **(F)** YLY1, 2014. For *X*-axis: TL, tillering; SE, stem elongation; PI, panicle initiation; BT, booting; HD, heading.

For Japonica and Indica rice cultivars in Experiments 1, 3, 4, and 7, a higher N rate resulted in a higher PNC (**Figure 1**), although PNC declined during advancing maturity (TL to HD). For any particular growth stage, in any of the growing seasons and rice ecotypes, PNC varied substantially in response to the different N fertilizer rates. There was maximum variation in PNC among both ecotypes at TL, and minimum variation at the HD stage in each growing season. Under the varied N rates, PNC ranged from 2.29 to 3.21 (**Figures 1B,E**) and 1.06 to 1.82 (**Figures 1B,E**), respectively, at TL and HD stages for Japonica rice cultivars, while they were 2.25– 3.23 (**Figures 1D,F**) and 1.34–1.91 (**Figures 1C,D**) for Indica rice cultivars. Under N0 treatment Japonica rice cultivars showed higher PNC (2.45 and 1.35) (**Figure 1E**) than Indica rice cultivars (2.36 and 1.34) (**Figure 1F**) at TL and HD stages, respectively. In contrast, under the highest applications of N fertilizer (the N4 or N5 treatments, **Table 1**) Japonica rice cultivars showed lower PNC (3.21 and 31.82) (**Figure 1E**) than Indica rice cultivars (3.23 and 1.91) (**Figures 1D,F**) at TL and HD stages, respectively.

### Changes in Chlorophyll Meter Readings Over Growth Stages under Varied N Rates in Japonica and Indica Rice

**FIGURE 2:**
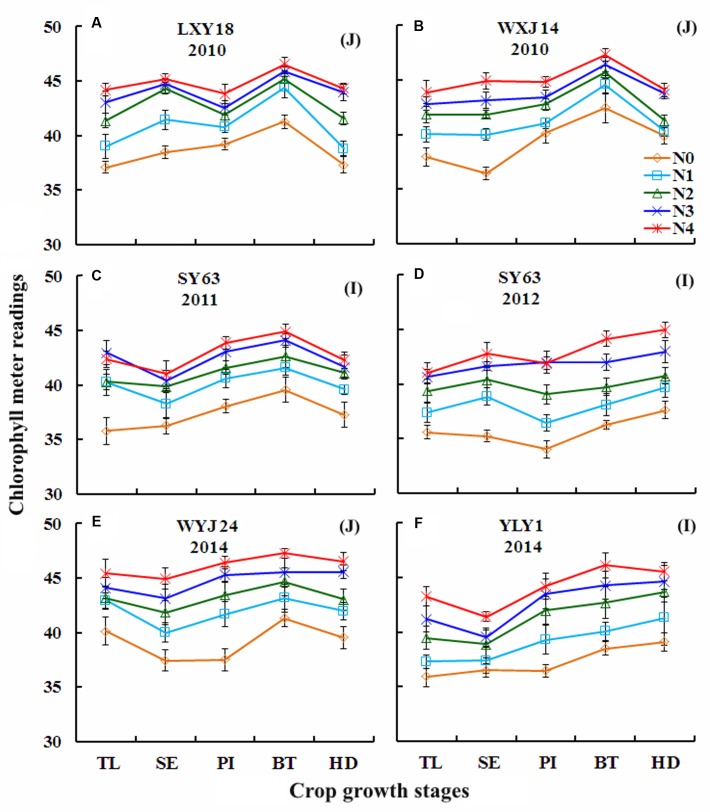
**Changes in chlorophyll meter reading over growth stages under varied N rates in Japonica (J) and Indica (I) cultivars during 2010–2014.** **(A)** LXY18, 2010; **(B)** WXJ14, 2010; **(C)** SY63, 2011; **(D)** SY63, 2012; **(E)** WYJ24, 2014; **(F)** YLY1, 2014. For *X*-axis: TL, tillering; SE, stem elongation; PI, panicle initiation; BT, booting; HD, heading.

A higher N fertilizer rate generally resulted in a higher chlorophyll meter readings in both rice ecotypes during vegetative growth period in Experiments 1, 3, 4, and 7 (**Figure 2**). Chlorophyll meter readings under varied N rates ranged from 37.10 to 45.4 (**Figures 2A,E**) and 37.30 to 46.6 (**Figures 2C,E**), respectively, at TL and HD stages for Japonica rice, while they were 35.6–43.3 (**Figures 2D,F**) and 37.3–45.5 (**Figures 2C,F**) for Indica rice. At TL and HD stages, Japonica rice showed higher chlorophyll meter readings [40.1, 45.4 (**Figure 2E**) and 39.9, 46.6 (**Figures 2B,E**)] than Indica rice [35.9, 43.3 (**Figure 2F**) and 39.1, 45.5 (**Figure 2F**)] under the lowest (N0, **Table 1**) and highest (N4 or N5) N application treatments, respectively. The maximum chlorophyll meter readings were observed at BT stages in all the experiments, irrespective of growing seasons, N rates and rice cultivars. Chlorophyll meter readings observed at BT stages under N0 and N4 treatments ranged from 41.2 to 47.3 (**Figures 2A,B**) and 38.5 to 46.1 (**Figure 2F**) in Japonica and Indica rice cultivars, respectively.

### Quantitative Relationships between Chlorophyll Meter Readings and PNC

**FIGURE 3:**
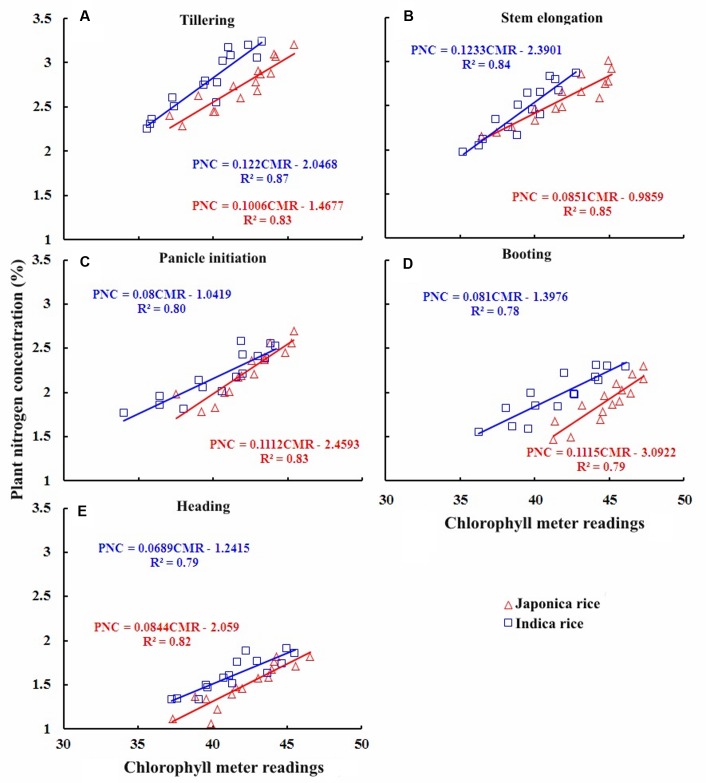
**Quantitative relationships between chlorophyll meter reading and plant nitrogen concentration (PNC) at different growth stages in Japonica and Indica rice.** **(A)** Tillering, **(B)** Stem elongation, **(C)** Panicle initiation, **(D)** Booting, **(E)** Heading.

The statistical analyses of results from Experiments 1, 3, 4, and 7 showed that PNC of Japonica and Indica rice cultivars were significantly and positively related with chlorophyll meter readings (pooled data of four top leaves) at TL, SE, PI, BT, and HD stages (**Figure 3**). The coefficients of determinations (*R*^2^) for the linear relationships between chlorophyll meter readings and PNC ranged from 0.79 to 0.85 and 0.78 to 0.87 over five growth stages in Japonica and Indica rice, respectively. The strongest relationship for Indica rice was observed at TL stages while in Japonica rice it was observed on SE stage. Overall, the relationships between chlorophyll meter readings and PNC satisfactorily explained the variation in rice PNC at different growth stages, suggesting that chlorophyll meter readings could be used to estimate plant N status in different ecotypes of rice.

Chlorophyll meter readings and PNC relationships of two rice ecotypes showed differences during vegetative growth period: the slope and intercept of the relationships for Indica rice were greater than Japonica rice (AT and MT) while the slopes of the relationships for Japonica rice were greater than Indica rice at SE, BT and HD stages. These differences were associated with varied ontogenetic responses to PNC due to genetic differences between the rice ecotypes. The dissimilarity in ontogenetic responses to PNC in two rice ecotypes was associated with differences in NRE.

### Quantitative Relationships between Chlorophyll Meter Readings and NNI

**FIGURE 4:**
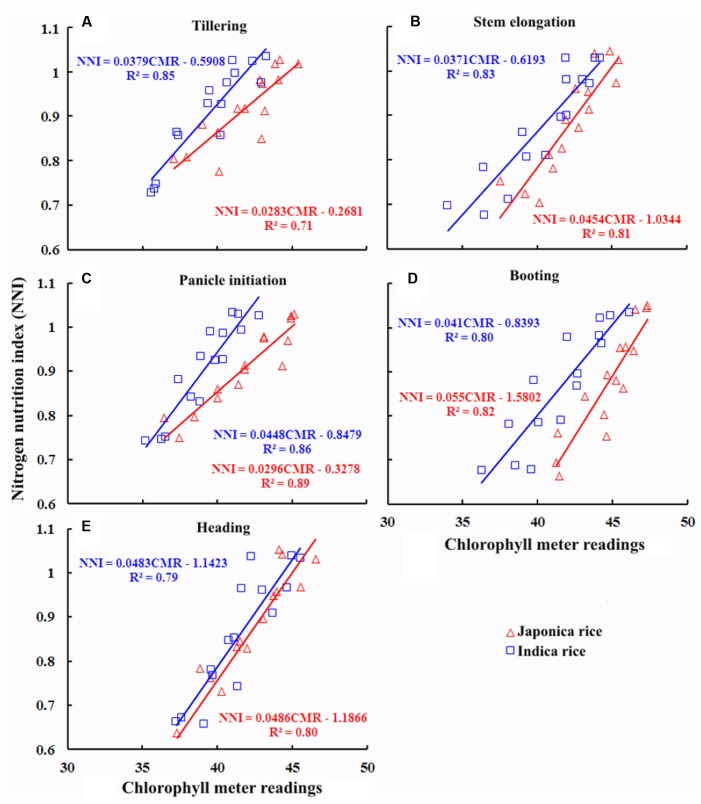
**Quantitative relationships between chlorophyll meter reading and nitrogen nutrition index (NNI) at different growth stages in Japonica and Indica rice.** **(A)** Tillering, **(B)** Stem elongation, **(C)** Panicle initiation, **(D)** Booting, **(E)** Heading.

Taking into account all the results obtained from Experiments 1, 3, 4, and 7, there were highly positive relationships between chlorophyll meter readings and NNI at different growth stages (**Figure 4**). These relationships were expressed by a linear function during the course of growing season. The *R*^2^ values ranged from 0.71 to 0.89 and 0.79 to 0.86 during crop growth period in Japonica and Indica rice, respectively. The strongest relationships for both ecotypes of rice were observed at PI stage. The close relationships between chlorophyll meter readings and NNI based on whole plant N concentration seemed consistent with the positive relationship at different growth stages, hence, can be effectively elucidated the changes in NNI across the vegetative growth period in different ecotypes of rice. The algorithms could be used for in-season estimation of NNI for quantifying the crop N status. The relationships between chlorophyll meter readings and NNI of two rice ecotypes exhibited inconsistencies during vegetative growth period, except for HD stages where similar slope and intercept of the relationship were observed for both ecotypes. These inconsistencies in the relationships are likely to have been caused by genetic differences between the rice ecotypes that resulted in diverse ontogenetic responses to NNI. The differences between the intercept of the relationships toward advancing maturity in two rice ecotypes were due to decline in PNC.

### Quantitative Relationships between Chlorophyll Meter Readings and AND

**FIGURE 5:**
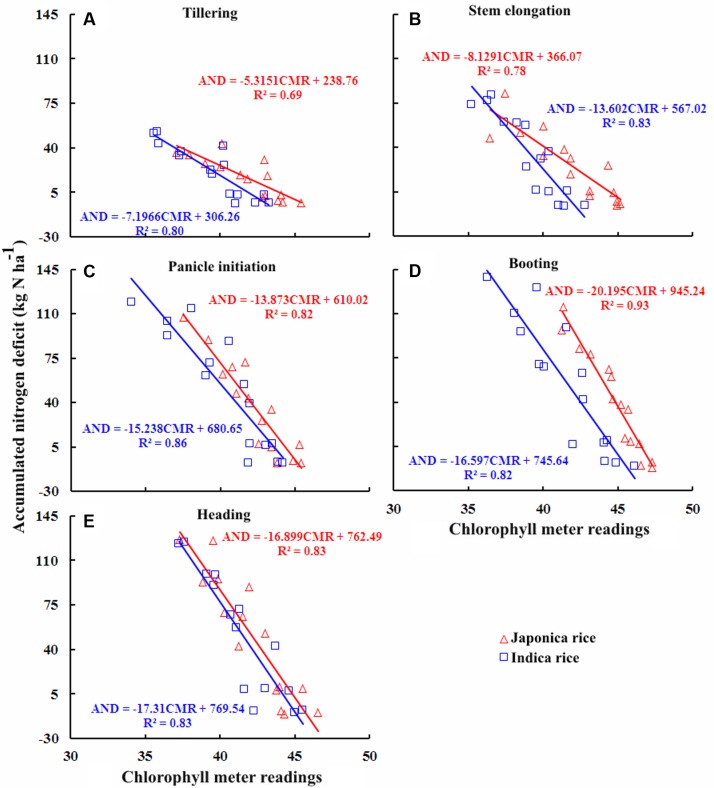
**Quantitative relationships between chlorophyll meter reading and accumulated nitrogen deficit (AND) at different growth stages in Japonica and Indica rice.** **(A)** Tillering, **(B)** Stem elongation, **(C)** Panicle initiation, **(D)** Booting, **(E)** Heading.

The relationships between chlorophyll meter readings and AND at TL, SE, PI, BT, and HD stages were established for in-season estimation of AND during vegetative growth period in Japonica and Indica ecotypes of rice, (**Figure 5**). AND was linearly related to chlorophyll meter readings during the course of growing season. Chlorophyll meter readings and AND at different growth stages were strongly negatively correlated. The *R*^2^ values were ranged from 0.69 to 0.93 and 0.80 to 0.86 for five growth stages in Japonica and Indica rice, respectively. The strongest relationships were observed at PI for Indica rice and at BT stage for Japonica rice. The slope of the relationships between chlorophyll meter readings and AND in Japonica and Indica rice varied during different growth stages. At AT, MT, and SE, the slope of the relationships for Japonica rice was greater than that for Indica rice. During BT stage the slope of Indica rice was greater than Japonica rice. However, at HD stage the slopes of both rice ecotypes were equivalent. These divergences were linked with varied ontogenetic responses due to genetic variances between the rice ecotypes. The variances between the intercept of the models with increasing AND to increase in DM accumulation were attributed to genetic as well as NRE differences between two rice ecotypes.

### Quantitative Relationships between Chlorophyll Meter Readings and NR

**FIGURE 6:**
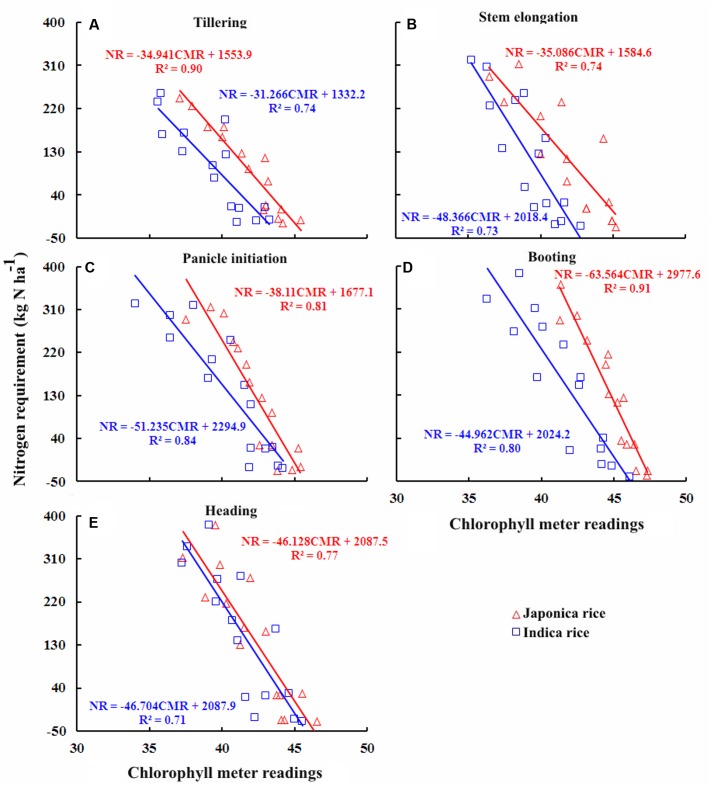
**Quantitative relationships between chlorophyll meter reading and nitrogen requirement (NR) at different growth stages in Japonica and Indica rice.** **(A)** Tillering, **(B)** Stem elongation, **(C)** Panicle initiation, **(D)** Booting, **(E)** Heading.

Quantitative relationships between leaf chlorophyll meter readings and plant NR were calculated from the data acquired from experiments conducted during 2010–2014 in order to better understand the relationships between leaf chlorophyll meter readings and plant NR during vegetative growth period in Japonica and Indica rice cultivars (**Figure 6**). The NR was expressed as a function of chlorophyll meter readings at TL, SE, PI, BT, and HD stages in different rice ecotypes. The NR at different growth stages showed a highly negative relationship to chlorophyll meter readings, with *R*^2^ values greater than 0.74–0.91 and 0.71–0.84 for Japonica and Indica rice, respectively. The strongest relationships were observed at PI for Indica rice and at the BT stage for Japonica rice. Overall, these relationships well explained the variation in NR of rice ecotypes at different crop growth stages. Chlorophyll meter readings and NR relationships of rice ecotypes displayed incongruities throughout the vegetative growth period where the slope of the relationships differs substantially from each other; however, at HD stage the slope of the relationships was similar for Japonica and Indica rice. These inconsistencies were related with varied ontogenetic responses due to genetic differences between the rice ecotypes. The differences between the intercept of the relationships between DM and NR were ascribed to genetic as well as NRE variances between two rice ecotypes.

### Validation of the Quantitative Relationships between Chlorophyll Meter Readings and N Parameters

**FIGURE 7:**
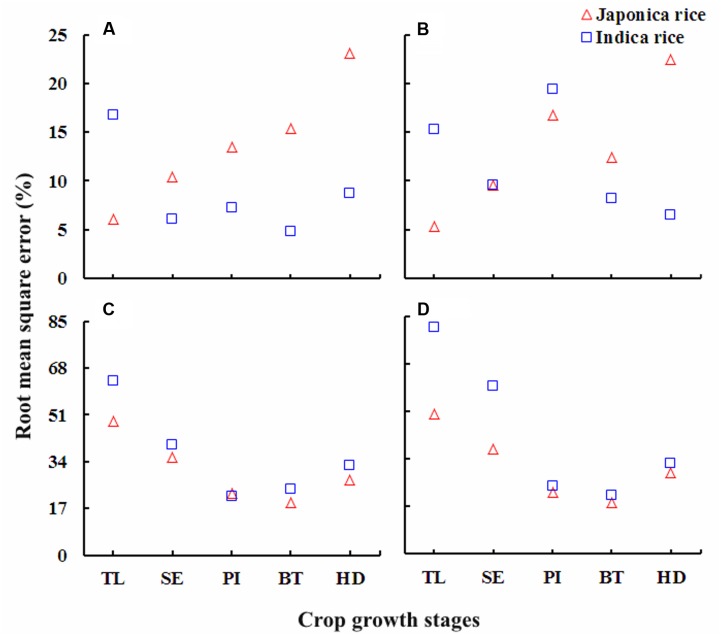
**The RMSE (%) values for the validation of the quantitative relationships between,** **(A)** chlorophyll meter reading and plant nitrogen concentration (PNC), **(B)** nitrogen nutrition index (NNI), **(C)** accumulated nitrogen deficit (AND) and **(D)** nitrogen requirement (NR) at different growth stages in Japonica and Indica rice. For *X*-axis: TL, tillering; SE, stem elongation; PI, panicle initiation; BT, booting; HD, heading.

The performance of the relationships between chlorophyll meter readings and PNC, NNI, AND, and NR in Japonica and Indica ecotypes of rice at different growth stages derived on data from Experiments 1, 3, 4, and 7 were validated for each growth stage on the independent data sets acquired from Experiments 2, 5, and 6. Generally, the validation relationships for PNC, NNI, AND, and NR as a function of chlorophyll meter readings at different crop growth stages for both ecotypes of rice showed a similar level of variance to the original relationships, although the variance differed with growth stages and N parameters (**Figure 7**).

## Discussion

### Variability in Plant N Concentration and Chlorophyll Meter Readings

The trend for decreased PNC across the different N rates, growth stages, cultivars/ecotypes and growing seasons in this study were consistent with the previous reports on wheat (Justes et al., 1994), barley (Zhao et al., 2016b) and rice (Ata-Ul-Karim et al., 2013). This variability toward advancing maturity could be attributed to a decline in the fraction of total plant N associated with photosynthesis (Bélanger and Richards, 2000), change in leaf/stem ratio and self-shading of leaves (Yue et al., 2012). The phenomenon of gradually decreasing gradient in PNC increased from the top to the bottom of the plant canopy toward advancing maturity and could be well justified by the mechanism of plant N dilution process within the plant canopy (Gastal et al., 2014). Moreover, N recycling associated with light distribution in plant canopy is in accordance with the canopy photosynthesis optimization theory (Hirose and Werger, 1987). The differences in variation of PNC at TL and HD stages between two ecotypes of rice under N-limiting and non-N-limiting growing conditions were attributed to the genetic ability to respond the varying growth conditions and were in agreement with previous reports on rice (Yoshida et al., 2006; Ata-Ul-Karim et al., 2013).

The variability of chlorophyll meter readings across the varied N rates, growth stages, cultivars/ecotypes and growing seasons in rice showed similar trends as reported in previous studies in rice and barley (Yang et al., 2014; Yuan et al., 2016a; Zhao et al., 2016b). At early growth stages (TL), the chlorophyll meter readings generally showed non-significant differences across the ecotypes and N application rates. This result was attributed to the large amount of residual soil N at transplanting (Peng et al., 1996), which resulted in lower N restriction at early growth stages. The differences in chlorophyll meter readings at different growth stages under varied N rates might be attributed to the genetic differences (specific weight and leaf thickness) between the two ecotypes. The higher chlorophyll meter readings in both rice ecotypes at BT stage were due to the supplemental N application and were in consensus with earlier studies on rice (Yang et al., 2014).

### Relationships between Chlorophyll Meter Readings and PNC, NNI, AND, and NR

The higher *R*^2^ values for the relationships between chlorophyll meter readings and PNC, NNI, AND, and NR across the ecotypes and growth stages suggested that chlorophyll meter readings could be reliably used for in-season estimation of PNC and critical N parameters in both Japonica and Indica rice. The positive relationships observed between chlorophyll meter readings and both parameters PNC and NNI in this study were consistent with previously established relationships between chlorophyll meter readings and both PNC and NNI in corn (Wood et al., 1992). In contrast, the relationships between chlorophyll meter readings and both AND and NR were negative. This study to develop these relationships. The strongest relationships between chlorophyll meter readings and PNC at early growth (TL and SE) stages, while the gradually and relatively weakening relationships toward advancing maturity (BT and HD) in both rice ecotypes confirm the N dilution process in plant canopy and were in consensus with previous reports (Justes et al., 1994; Ziadi et al., 2010). The strongest relationships between chlorophyll meter readings and NNI, AND, and NR at PI and BT stages in two rice ecotypes were related to higher crop growth and N uptake rates. The differences between the relationships of two rice ecotypes were attributed to their genotypic differences (Weng and Chen, 1987). The critical N parameters are reliable and precise indicators to characterize the plant N status of the rice crop throughout the growth period. It was suggested that the critical N parameters could be used as a reference with simpler methods such as chlorophyll meter to determine crop N status (Bélanger et al., 2003). Although attempts have been made to relate the chlorophyll meter readings with PNC and NNI using several indirect methodologies, such as relative chlorophyll meter readings (Ziadi et al., 2008), chlorophyll meter readings of different leaf position (Prost and Jeuffroy, 2007; Yang et al., 2014; Zhao et al., 2016b) and positional difference indices for estimating crop N status (Zhao et al., 2016b), there remains contradiction regarding the effect of different leaf positions on chlorophyll meter readings, which affects the reliability of these techniques in estimating plant N status. A recent report on rice by Wang et al. (2014) used the averaged chlorophyll meter readings of top four fully expanded leaves and integrated chlorophyll meter readings with canopy color related indices acquired using a digital still camera for in-season N diagnosis in rice crop, which has potential to minimize the influences of leaf position on the plant and sampling location on the leaf on chlorophyll meter readings. Yet, the techniques required for image processing makes this approach complex as compared to that developed in the present study. Moreover, the relationships between chlorophyll meter readings and canopy color related indices were developed using the data acquired from two experiments in the same growing season, location and ecotype, which may affect their applicability at different locations and ecotypes grown under different climatic conditions (even at the same site in different growing seasons). In contrast, the methodology developed in the present study is simpler as compared to the existing ones and has the potential to minimize the controversies attributed due to growth stage, cultivar type, specific leaf weight, leaf position on the plant, and sampling location on a leaf. Moreover, as compared to the relationships between chlorophyll meter readings and canopy color related indices, the relations developed in the present study were well justified by the mechanism of plant N dilution within the plant canopy (Gastal et al., 2014) and canopy photosynthesis optimization theory (Hirose and Werger, 1987). Additionally, the relationships were validated using the data acquired from different locations, ecotypes and years. Therefore, the relations developed in the present study were comparatively more reliable and can be used for rapid, in-expensive and robust in-season estimation of PNC and critical N parameters at different growth stages in rice ecotypes. The differences observed in the relationships between chlorophyll meter readings and PNC, NNI, AND, and NR for the two rice ecotypes (Japonica and Indica) in present were in agreement with the previous report which stated that Japonica rice differs from Indica rice in genetic makeup, crop growth rate, ontogenesis, and N uptake (Weng and Chen, 1987; Yoshida et al., 2006; Ata-Ul-Karim et al., 2013). The differences between the slope and intercept responses of the relationship at different growth stages of two rice ecotypes were likely to be caused by the aforementioned factors, which further resulted in variation of DM partition and translocation of photosynthates between two rice ecotypes (Ata-Ul-Karim et al., 2017).

### Applicability of Chlorophyll Meter Readings for Assessing Plant N Status

The chlorophyll meter readings generally differed among sites, cultivars and seasons while several other factors such as growth stage, leaf position on plant and sampling location on leaf could effect the chlorophyll meter readings. However, the results of present study confirmed our hypothesis that the averaged values of chlorophyll meter readings of four uppermost fully expanded leaves for a cultivar and then for an ecotype could considerably minimize the influences of factors affecting the chlorophyll meter readings and were in consensus with the previous report on rice (Wang et al., 2014). The destructive sampling prerequisite for assessment of critical N parameters may limit the practical execution of this methodology, yet the latest precision management tools such as the GreenSeeker and Crop Circle ACS-470 sensor can be effectively used for reliable in-season estimation of the critical N parameters at different phenological stages (Mistele and Schmidhalter, 2008; Ata-Ul-Karim et al., 2016, 2017). Chlorophyll meter readings can also be integrated with canopy color related indices obtained by the digital still color camera for in-season N diagnosis in rice crop. However, due to techniques involved in image processing, this approach is complex as compared to that developed in the present study. Moreover, previous studies have reported that chlorophyll meter readings can be effectively used as a reference for diagnosing crop N status using critical N parameters (Bélanger et al., 2003). The relationships established in the present study by integrating chlorophyll meter readings and critical N parameters indicated that the chlorophyll meter readings could be implemented for reliable, rapid, and cost-effective estimation of in-season plant N status during the vegetative growth period in different rice ecotypes. Overall the performance of the relationships between chlorophyll meter readings and PNC, NNI, AND, and NR to estimate the in-season plant N status was robust enough across the growth stages. However, the relationships of chlorophyll meter readings and critical N parameters, especially the chlorophyll meter readings, AND and NR were more reliable. These relationships were strongest at PI and BT stages, the stages that cover 65–75% of the rice growth period and rice plants have generally experienced most of the growth limiting factors at these crop growth stages (Chang et al., 2005; Ata-Ul-Karim et al., 2016). The newly developed integrated approach has promise as a precision N management methodology in rice cropping systems. Although the integration of chlorophyll meter readings and critical N parameters showed robust relationships in the present study, investigations using various N management practices, cultivars/ecotypes in different cropping systems will further prove this integrated approach as a reliable diagnostic tool for its widespread use in diverse rice production systems.

For the first time, the current study established a comprehensive integration of chlorophyll meter readings and different critical N parameters in Japonica and Indica rice ecotypes for developing a rapid, non-destructive, inexpensive and eco-friendly N management plan. Our results showed that the higher N fertilizer application rate generally resulted in a higher PNC and chlorophyll meter readings in both ecotypes of rice during the vegetative growth period, and chlorophyll meter readings can be used to for reliable estimation of in-season PNC and critical N parameters

## Author Contributions

SA, YZ, and WC conceived the idea and led the study design. SA carried out the experiment, performed analysis and wrote the paper. QC, LT, and MR assisted with study design and experiments. SA and WC edited the manuscript.

## Conflict of Interest Statement

The authors declare that the research was conducted in the absence of any commercial or financial relationships that could be construed as a potential conflict of interest.
